# Physical activity levels determine exercise-induced changes in brain excitability

**DOI:** 10.1371/journal.pone.0173672

**Published:** 2017-03-09

**Authors:** Tea Lulic, Jenin El-Sayes, Hunter J. Fassett, Aimee J. Nelson

**Affiliations:** Department of Kinesiology, McMaster University, Hamilton, Canada; University Medical Center Goettingen, GERMANY

## Abstract

Emerging evidence suggests that regular physical activity can impact cortical function and facilitate plasticity. In the present study, we examined how physical activity levels influence corticospinal excitability and intracortical circuitry in motor cortex following a single session of moderate intensity aerobic exercise. We aimed to determine whether exercise-induced short-term plasticity differed between high versus low physically active individuals. Participants included twenty-eight young, healthy adults divided into two equal groups based on physical activity level determined by the International Physical Activity Questionnaire: low-to-moderate (LOW) and high (HIGH) physical activity. Transcranial magnetic stimulation was used to assess motor cortex excitability via motor evoked potential (MEP) recruitment curves for the first dorsal interosseous (FDI) muscle at rest (MEP_REST_) and during tonic contraction (MEP_ACTIVE_), short-interval intracortical inhibition (SICI) and facilitation (SICF), and intracortical facilitation (ICF). All dependent measures were obtained in the resting FDI muscle, with the exception of AMT and MEP_ACTIVE_ recruitment curves that were obtained during tonic FDI contraction. Dependent measures were acquired before and following moderate intensity aerobic exercise (20 mins, ~60% of the age-predicted maximal heart rate) performed on a recumbent cycle ergometer. Results indicate that MEP_REST_ recruitment curve amplitudes and area under the recruitment curve (AURC) were increased following exercise in the HIGH group only (p = 0.002 and p = 0.044, respectively). SICI and ICF were reduced following exercise irrespective of physical activity level (p = 0.007 and p = 0.04, respectively). MEP_ACTIVE_ recruitment curves and SICF were unaltered by exercise. These findings indicate that the propensity for exercise-induced plasticity is different in high versus low physically active individuals. Additionally, these data highlight that a single session of aerobic exercise can transiently reduce inhibition in the motor cortex regardless of physical activity level, potentially priming the system for plasticity induction.

## Introduction

Regular aerobic exercise can influence cellular [1–6] and molecular [5, 7, 8] processes thereby altering hippocampal and sub-cortical loci [1–6] as well as increasing levels of key neurotrophic factors, such as brain derived neurotrophic factor (BDNF) [7], that mediate neuroplasticity [9]. Further, executive function, such as response inhibition and processing speed, is enhanced in physically active individuals [1, 2, 5, 10, 11]. Thus, long-term exercise appears to facilitate cognitive function and memory through neuroplasticity and neuroprotective mechanisms. Additionally, regular exercise leads to chronic physiological adaptions that impact the response to a single session of aerobic exercise (see review: [12]). These adaptions include increased metabolic efficiency at a given heart rate [12], increased stroke volume [13], increased brain perfusion [14], and alterations in individual responses to stress, anxiety, and perceived rate of exertion [12]. As such, individuals who regularly exercise may respond differently to a single session of exercise than those who do not.

A single session of aerobic exercise has the capacity to induce short-term neuroplasticity within the human motor cortex, as assessed via cortical circuits evoked by transcranial magnetic stimulation (TMS). For example, intracortical facilitation (ICF) increases [15] while short-interval intracortical inhibition (SICI) decreases [15, 16] or does not change [17] following a single session of exercise. ICF is thought to reflect N-methyl_D_-aspartate (NMDA) receptors [18], while SICI is mediated via GABA_A_ receptor activity [19, 20]. Both ICF and SICI have been implicated in cortical reorganization and plasticity within the primary motor cortex [15, 21]. Further, motor evoked potentials (MEPs), a measure of corticospinal excitability [22, 23], are unchanged following a single session of aerobic exercise in individuals who are relatively sedentary [15, 16]. Aerobic exercise, therefore, provides an opportunity to create short-term changes in specific TMS circuits that may serve as targets for promoting neuroplasticity. Finally, a single session of aerobic exercise can be used to supplement other plasticity inducing approaches to yield greater effects [24–26].

MEPs can be obtained in the resting or actively contracted muscle to assess distinct mechanisms of corticospinal excitability [22, 23]. To date, no studies have investigated whether exercise induces short-term changes in MEPs in actively contracted muscles. Compared to relaxed muscle, voluntarily activation reduces the threshold for TMS activation of motor neurons [23]. Therefore, quantifying MEPs in the active versus resting state assesses corticospinal output with and without the voluntary activation of motor neurons. For example, MEPs are reduced in individuals with spinal cord injury [27] and Parkinson’s disease (PD) [28] compared to controls when they are measured in the active but not resting muscle. Therefore, it is important to determine whether corticospinal excitability can be modulated with aerobic exercise via either of these mechanisms.

Short interval intracortical facilitation (SICF) is considered to reflect GABA_A_ receptor activity [29] and is facilitated in PD [30] and Fabry’s disease [31]. SICF is unchanged following repetitive TMS [32, 33]. However, the natural stimulus of aerobic exercise may have the capacity to alter SICF and operate as a method to create neuroplasticity in these populations. To date, no studies have investigated the impact of a single session of aerobic exercise on the SICF circuit, yet this information may benefit clinical neuroscience research.

The type of exercise and the population tested may influence the opportunity for exercise-induced short-term plasticity. First, aerobic exercise is favored as it increases BDNF more frequently than resistance training (see review [34]). Second, physical activity levels influence the propensity for plasticity. For example, paired associative stimulation (PAS) induces short-term plasticity only in the corticospinal output of physically active individuals [35]. The effects of physical activity levels on exercise-induced plasticity have yet to be investigated.

In the present study, we aimed to identify the TMS circuits that are modulated following a single session of aerobic exercise and to determine if physical activity levels influence the magnitude of exercise-induced plasticity in these circuits. To assess plasticity, we measured TMS-evoked circuits including MEP recruitment curves in the resting and active states, SICI, ICF, and SICF in a resting hand muscle before and after aerobic exercise. To our knowledge, this is the first study to investigate the influence of physical activity levels on exercise-induced plasticity in these circuits. Our data indicate that a single session of aerobic exercise induces changes in resting MEP recruitment curves, SICI, and ICF, confirming the capability for exercise to induce short-term plasticity. These findings suggest that aerobic exercise, as a plasticity-inducing paradigm, has differential effects on corticospinal excitability depending on physical activity level.

## Methods

### Participants

Twenty-eight young adults who self-identified as physically active (HIGH: N = 14, 22.1 ± 2.8 years, 9 female) or sedentary (LOW: N = 14, 20.6 ± 0.84, 8 female) participated in this study. All participants were right-hand dominant as determined by the modified version of the Edinburgh Handedness Scale [36] and had no known history of neurological disease. Participants were screened for contraindications to TMS [37] and exercise using the Physical Activity Readiness Questionnaire (PAR-Q) [38]. Written informed consent was obtained prior to participation. This study was approved by the McMaster Research Ethics Board and conformed to the Declaration of Helsinki.

Physical activity levels were determined using the International Physical Activity Questionnaire (IPAQ) that quantifies the physical activity performed in the past week [39]. Participants who accumulated more than 3000 metabolic equivalents (METs) on IPAQ were considered highly physically active (HIGH), while those who accumulated less than 3000 METs were considered low-to-moderately active (LOW) [16, 40]. The IPAQ scores were significantly higher for the HIGH (METs 7631 ± 6120; 3231–21162) versus LOW (METs 1305 ± 773; 360–2892) group (p < 0.001). To verify the grouping of participants in the HIGH and LOW categories, the Minnesota Leisure Activity Questionnaire [41] was used to assess long term physical activity levels in each participant. These data confirm the division of HIGH (7441 ± 6157) and LOW (965 ± 1062) activity levels (p < 0.001) in the sample tested.

### Electromyography (EMG) recording

Electromyography (EMG) was recorded using surface electrodes (9 mm diameter Ag-AgCl) over first dorsal interosseous (FDI) muscle of the right hand in a belly tendon montage. A wet ground electrode was placed around the forearm. EMG measurements were amplified (x1000), and filtered with a band pass (20 Hz—2.5 kHz) (Intronix Technologies Corporation Model 2024F with Signal Conditioning; Intronix Technologies Corporation, Bolton, Canada) and subsequently digitized at 5 kHz (Power1401, Cambridge Electronic Design, Cambridge, UK). EMG data was collected using Signal software version 6.02 (Cambridge Electronic Design, Cambridge, UK).

### Maximum Voluntary Contraction (MVC)

Participants completed three maximal isometric contractions of the FDI against an immovable structure. Each contraction persisted for 5 s with a 30 s rest interval between trials. The largest maximum EMG activity obtained from the three trials was deemed the maximum voluntary contraction (MVC) of FDI for a given participant. The level of EMG corresponding to the MVC was displayed to the participant on an oscilloscope. The voltage that corresponded to 10% of MVC was calculated and displayed on the oscilloscope with a horizontal target line. The participant then performed a contraction of FDI to move a second horizontal line controlled by their EMG to match the position of the target line. Therefore, participants used their own visual feedback to maintain the 10% MVC during the acquisition of active motor threshold and active MEP recruitment curve (see below).

### Transcranial Magnetic Stimulation (TMS)

Single and paired monophasic TMS pulses were delivered using a custom-built 50 mm diameter figure-of-eight branding coil connected to a Magstim Bistim stimulator (Magstim, Whitland, UK). The TMS coil was placed over the optimal location to elicit MEPs in the relaxed right FDI. Coil was positioned 45 degrees in relation to the parasagittal plane to induce posterior-to-anterior current in the cortex. The motor hotspot for FDI muscle of the right hand was determined within the left hemisphere motor cortex and defined as the location that consistently elicited MEPs in the relaxed FDI muscle. The motor hotspot was marked by digital registration using a standard MRI template via Brainsight Neuronavigation (Rogue Research, Canada). Resting motor threshold (RMT) and active motor threshold (AMT) were determined for the right FDI. RMT was defined as the lowest intensity required to evoke an MEP ≥ 50 μV in 5 out of 10 consecutive trials in relaxed FDI muscle [37]. AMT was defined as the lowest intensity required to evoke an MEP ≥ 200 μV in 5 out of 10 consecutive trials while participants maintained ~10% of their MVC in right FDI [37]. Visual feedback of the right FDI contraction was provided using an oscilloscope.

### Motor Evoked Potential (MEP) recruitment curves

MEP recruitment curves were obtained in the resting and active (~10% MVC) right FDI muscle. For each curve, three stimuli were delivered at each of 90%, 100%, 110%, 120%, 130%, 140% and 150% RMT or AMT in a randomized order. The number of pulses delivered at each intensity reflects the results of recent studies examining the consistency of MEPs with few stimuli [42, 43].

### Short-interval Intracortical Inhibition (SICI) and Intracortical Facilitation (ICF)

SICI and ICF were investigated using interstimulus intervals (ISI) of 2 ms and 10 ms between the conditioning stimulus (CS) and test stimulus (TS), respectively. Each circuit was tested with CS intensities of 80% and 90% AMT and with TS set to evoke MEPs with peak-to-peak amplitudes of ~1 mV in the right FDI at rest. For each circuit, 20 trials were acquired whereby equal numbers of unconditioned (i.e. MEP_TS_) and conditioned (MEP_CS-TS_) trials were randomly delivered.

### Short-interval Intracortical Facilitation (SICF)

SICF was measured using a similar method to that described by Ziemann et al. [44]. SICF was investigated using ISIs of 1.2 ms and 2.5 ms and was recorded in two blocks of 20 trials for each ISI (20 at 1.2 ms, 20 at 2.5 ms; 10 TS and 10 CS/TS each block). CS intensity was set to 90% RMT for both ISIs. The TS intensity was set to evoke MEPs with peak-to-peak amplitudes of ~1 mV in the right FDI at rest.

### Experimental design

**Fig 1:**
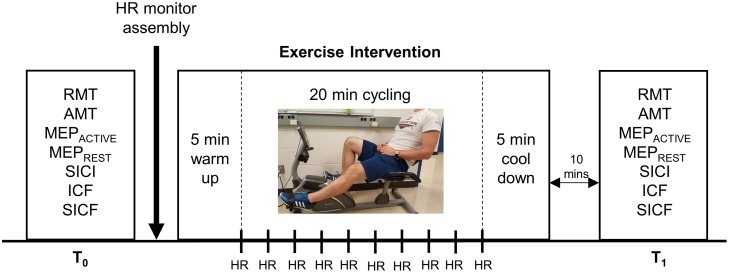
Experimental timeline. Measures of resting motor threshold (RMT), active motor threshold (AMT), motor evoked potential (MEP) recruitment curves obtained at rest (MEP_REST_) and during ~10% MVC (MEP_ACTIVE_), short-interval intracortical inhibition (SICI), intracortical facilitation (ICF) and short-interval intracortical facilitation (SICF) were acquired prior to (T_0_) and ten minutes following the cessation of exercise (T_1_).The order of dependent measure acquisition was pseudo-randomized across participants using a Williams Square design. The exercise intervention began following the assembly of the heart rate (HR) monitor and involved 5 minutes of cycling warm-up, 20 minutes of moderate-intensity exercise (50–70% of age-predicted maximal heart rate (HR)) and 5 minutes of cycling cool-down. During the 20-minute exercise, resistance was adjusted online to maintain HR in the target range. HR was recorded every 2 minutes as shown.

The experimental timeline is depicted in Fig 1. The session was divided into two time blocks: T_0_ (pre-exercise) and T_1_ (10 minutes post-exercise). All dependent measures were obtained in the resting FDI muscle, with the exception of AMT and MEP_ACTIVE_ recruitment curves that were obtained during tonic FDI contraction corresponding to ~10% MVC. The TMS intensity to elicit ~1 mV response in the resting FDI was re-assessed prior to the start of T_1_. The order of dependent measure acquisition was pseudorandomized (William Square Counterbalance).

### Exercise procedures

Participants completed a lower limb exercise on a recumbent cycle ergometer (Exerpeutic Heavy Duty Magnetic Recumbent Bike with Pulse, PARADIGM Health & Wellness, USA). The exercise session included 5 minutes of warm up at a comfortable pace, 20 minutes of moderate intensity exercise at 50–70% age-predicted maximal heart rate (i.e. 220 –age) [45], followed by 5 minutes of cool down at a comfortable pace. All individuals were maintained at ~60% of their age-predicted maximal heart rate, as performed elsewhere [15, 40]. Heart rate was monitored using an FT1 Polar heart rate monitor (FT1/FT2 watch and chest strap—Polar, Australia) and recorded by the experimenter every two minutes during the 20-minute exercise period. This information was used to adjust the resistance on the ergometer to maintain the targeted age-predicted heart rate range. Participants were instructed to maintain a cycling speed of 8–12 miles per hour, as indicated on the ergometer display available to them. Throughout the exercise, participants kept their arms comfortably relaxed in their lap to ensure that the FDI muscle, the target muscle for TMS-evoked measures, was not active during the exercise intervention.

### Data analysis and statistics

For MEP recruitment curves, the mean peak-to-peak amplitude was calculated for each RMT and AMT intensity (90–150%). All slopes were calculated in Microsoft Excel using linear regression of the entire recruitment curve and were subsequently tested for correlation with IPAQ scores. The area under the recruitment curve (AURC) was obtained by taking a trapezoidal integration of the recruitment curves. For paired-pulse TMS measures, the mean peak-to-peak MEP amplitude was calculated for the conditioned and unconditioned stimuli at each CS or ISI intensity separately. The percentage inhibition and facilitation was then calculated as a ratio of conditioned over unconditioned stimulus (CS-TS/TS). For each individual, the depth of the SICI and ICF was examined at T_0_ for each of the two CS intensities tested (80%, 90% AMT). The CS intensity at which the greatest depth was observed at T_0_ was chosen for further analyses. Since reductions in SICI were hypothesized from previous research [15, 16], participants were required to demonstrate a minimum of 5% SICI at T_0_ to be included in the SICI analysis. Additionally, since increases in facilitation were hypothesized [15] individuals were required to present with a minimum of 5% ICF or SICF at T_0_ to be included in the ICF or SICF analysis.

Group-level statistics included normality testing via the Shapiro-Wilk analysis. Outlier data were identified via SPSS, as 3 times above or below the interquartile range and were removed from further analyses. Specifically, one participant was removed from the HIGH group for resting and active MEP recruitment curves, and one participant was removed from the LOW group for SICI, ICF, and SICF_1.2ms_. Non-normally distributed data was ranked and a non-parametric Conover’s ANOVA was performed [46]. MEP recruitment curves were analyzed using three-way Conover’s ANOVA using within-subject factors TIME (2 levels: T_0_, T_1_) and factor INTENSITY (7 levels: 90, 100, 110, 120, 130, 140, 150% RMT/AMT) and between-subject factors GROUP (2 levels: LOW, HIGH). RMT, AMT, and SICI were analyzed using two-way ANOVA while AURC, ICF, and SICF were analyzed using a two-way Conover’s ANOVA with within-subject factor TIME (2 levels: T_0_, T_1_) and between-subject factor GROUP (2 levels: LOW, HIGH). For normally distributed dependent measures, post-hoc testing was performed using two-tailed t-tests. Post-hoc tests for non-parametric data included a Wilcoxon Signed-Rank for within group comparisons and Mann-Whitney U test for between group differences. All post-hoc testing was Bonferroni corrected. The significance level was set to p ≤ 0.05.

## Results

All participants successfully completed the study and performed the aerobic exercise at ~60% of their age-predicted maximal heart rate (LOW: 60.5 ± 1.9%, HIGH: 58.9 ± 3.3%, p = 0.154). Table 1 displays the results of all statistical analyses with associated effect sizes and 95% confidence intervals.

**Table 1:** Statistical analyses.

Dependent Measure	ANOVA
RMT	TIME _(1,26)_ = 0.101, p = 0.753
	GROUP_(1,26)_ = 0.042, p = 0.839
	**TIME x GROUP**_**(1,26)**_ **= 9.449, p = 0.005** ^#^
	L (N = 14): T_0_: 38.6 ± 1.62%MSO T_1_: 37.5 ± 1.79%MSO, *d* = 0.17
	H (N = 14): T_0_: 37.1 ± 1.93%MSO T_1_: 38.0 ± 2.09%MSO, *d* = 0.12
MEP_REST_ Amplitude*	TIME _(1,25)_ = 0.577, p = 0.455
	GROUP_(1,25)_ = 1.447, p = 0.240
	INTENSITY_(6,20)_ = 79.32, p = 0.000
	**TIME x GROUP**_**(1,25)**_ **= 4.788, p = 0.038**
	**H: T**_**0**_**<T**_**1**_ **p = 0.002, *d* = 0.17, 95% CI: -0.93 to 0.61**
	TIME x INTENSITY_(6,20)_ = 0.761, p = 0.609
	INTENSITY x GROUP_(6,20)_ = 0.798, p = 0.583
	TIME x GROUP x INTENSITY_(6,20)_ = 1.594, p = 0.201
MEP_REST_ AURC*	TIME _(1,26)_ = 0.178, p = 0.676
	GROUP_(1,26)_ = 3.914, p = 0.059
	**TIME x GROUP**_**(1,26)**_ **= 6.10, p = 0.02**
	**H: T**_**0**_**<T**_**1**_ **p = 0.044, *d* = 0.54, 95% CI: -1.27 to 0.23 (uncorrected)**
AMT	TIME _(1,26)_ = 1.204, p = 0.283
	GROUP_(1,26)_ = 0.278, p = 0.603
	TIME x GROUP_(1,26)_ = 1.873, p = 0.187
	L (N = 14): T_0_: 26.1 ± 0.84%MSO T_1_: 24.7 ± 0.96%MSO, *d* = 0.42
	H (N = 14): T_0_: 24.5 ± 1.31%MSO T_1_: 24.6 ± 1.45%MSO, *d* = 0.02
MEP_ACTIVE_ Amplitude*	TIME _(1,25)_ = 3.554, p = 0.071
	GROUP_(1,26)_ = 0.365 p = 0.551
	INTENSITY_(6, 20)_ = 55.17, p = 0.000
	TIME x GROUP_(1,25)_ = 0.001, p = 0.981
	TIME x INTENSITY_(6, 20)_ = 0.617, p = 0.714
	INTENSITY x GROUP_(6, 20)_ = 1.406, p = 0.261
	TIME x GROUP x INTENSITY_(6, 20)_ = 1.34, p = 0.286
MEP_ACTIVE_ AURC*	TIME _(1,26)_ = 0.084, p = 0.775
	GROUP_(1,26)_ = 0.453, p = 0.507
	TIME x GROUP_(1,26)_ = 0.44, p = 0.513
	L (N = 14): T_0_: 6.08 ± 0.67 AURC T_1_: 4.73 ± 0.52 AURC, *d* = 0.60
	H (N = 14): T_0_: 5.99 ± 1.01 AURC T_1_: 5.25 ± 0.76 AURC, *d* = 0.22
SICI	**TIME**_**(1,22)**_ **= 8.674, p = 0.007**
	**T**_**0**_**<T**_**1**_**, p = 0.007, *d* = 0.512, 95% CI: -1.08 to 0.07**
	GROUP_(1,22)_ = 0.168, p = 0.686
	TIME x GROUP_(1,22)_ = 0.380, p = 0.544
SICI TS	TIME_(1,23)_ = 0.036, p = 0.851
	L (N = 13): T_0_: 1.03 ± 0.04 mV T_1_: 1.13 ± 0.06 mV, *d* = 0.54
	H (N = 11): T_0_: 1.16 ± 0.03 mV T_1_: 1.06 ± 0.06 mV, *d* = 0.57
ICF*	**TIME**_**(1,22)**_ **= 5.268, p = 0.032**
	**T**_**0**_**>T**_**1**_**, p = 0.04, *d* = 0.71, 95% CI: -0.14 to 1.51**
	GROUP_(1,22)_ = 0.000, p = 0.983
	TIME x GROUP_(1,22)_ = 0.222, p = 0.642
ICF TS	TIME_(1,23)_ = 0.060 p = 0.808
	L (N = 12): T_0_: 1.05 ± 0.08 mV T_1_: 1.12 ± 0.09 mV, *d* = 0.25
	H (N = 12): T_0_: 1.20 ± 0.08 mV T_1_: 1.18 ± 0.06 mV, *d* = 0.07
SICF_1.2ms_ *	TIME _(1,24)_ = 3.681, p = 0.067
	GROUP_(1,24)_ = 1.677, p = 0.208
	**TIME x GROUP**_**(1,24)**_ **= 5.274, p = 0.031** ^#^
	L (N = 13): T_0_: 1.61 ± 0.14 mV T_1_: 1.65 ± 0.13 mV, *d* = 0.08
	H (N = 13): T_0_: 2.12 ± 0.22 mV T_1_: 1.76 ± 0.21 mV, *d* = 0.46
SICF_1.2ms_ TS	TIME_(1,25)_ = 1.579, p = 0.221
	L (N = 13): T_0_: 0.96 ± 0.08 mV T_1_: 0.96 ± 0.07 mV, *d* = 0.00
	H (N = 13): T_0_: 0.99 ± 0.06 mV T_1_: 1.16 ± 0.07 mV, *d* = 0.74
SICF_2.5ms_*	TIME_(1,22)_ = 1.247, p = 0.276
	GROUP_(1,22)_ = 0.604, p = 0.445
	TIME x GROUP_(1,22)_ = 0.455, p = 0.507
	L (N = 13): T_0_: 1.88 ± 0.21 mV T_1_: 1.91 ± 0.23 mV, *d* = 0.04
	H (N = 11): T_0_: 1.68 ± 0.14 mV T_1_: 1.53 ± 0.17 mV, *d* = 0.40
SICF_2.5ms_ TS	TIME_(1,25)_ = 0.042, p = 0.840
	L (N = 13): T_0_: 0.98 ± 0.06 mV T_1_: 1.04 ± 0.06 mV, *d* = 0.28
	H (N = 11): T_0_: 1.09 ± 0.09 mV T_1_: 1.05 ± 0.09 mV, *d* = 0.14

*Conover’s ANOVA (ranked data) and subsequent non-parametric post-hoc analyses.

^#^: post-hoc analyses did not pass Bonferroni corrections.

Bolded values indicate significance as shown. Means ± SE displayed. *d*: Cohen’s D, 95% CI: 95% confidence intervalof effect size, T_0_ (baseline), T_1_ (10 minutes post-exercise), L (LOW group), H (HIGH group).

### Thresholds and MEP recruitment curves

**Fig 2:**
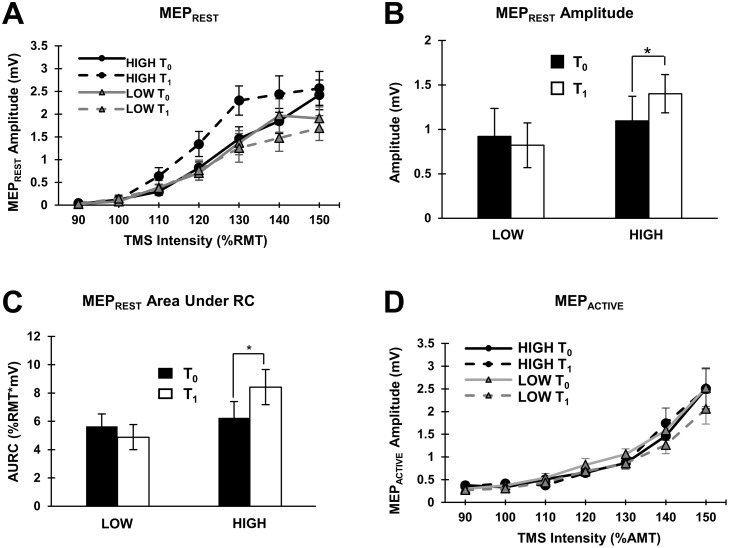
Thresholds and recruitment curves. **(A)** Group-averaged MEP_REST_ recruitment curves (with standard errors) at T_0_ and T_1_ for the LOW (N = 14) and HIGH (N = 13) groups. TMS intensity is defined as the percentage of RMT. Solid and dashed lines indicate pre (T_0_) and post (T_1_) values, respectively. **(B)** Histograms displaying TIME x GROUP interaction for group-averaged MEP_REST_ amplitude (with standard error; LOW: N = 14, HIGH: N = 13). The asterisk indicates a significant increase in MEP_REST_ amplitudes. **(C)** Histograms displaying TIME x GROUP interaction for group-averaged MEP_REST_ AURC (with standard error; LOW: N = 14, HIGH: N = 14). The asterisk indicates a significant increase in MEP_REST_ AURC. **(D)** Group-averaged MEP_ACTIVE_ recruitment curves (with standard errors) at T_0_ and T_1_ for the LOW (N = 14) and HIGH (N = 13) group. TMS intensity is defined as the percentage of the active motor threshold (AMT). Solid and dashed lines indicate pre (T_0_) and post (T_1_) values, respectively.

All participants were included in RMT analysis (N = 14 per group). The group-averaged RMT showed no significant differences among the means (Table 1). Thirteen and fourteen individuals were included in MEP_REST_ analysis for the HIGH and LOW groups, respectively. Group-averaged MEP_REST_ recruitment curves (with standard errors) are shown in Fig 2A and analyses revealed a significant TIME x GROUP interaction (p = 0.038; Table 1) such that amplitudes were significantly increased in the HIGH group following exercise (Wilcoxon: p = 0.002; Fig 2B). This effect was observed in nine individuals, with little to no change in three individuals and a decrease in MEPs following exercise in one individual. MEP_REST_ AURC (N = 14 per group) also revealed a significant TIME x GROUP interaction (p = 0.02; Table 1) such that AURC increased following exercise in the HIGH group only (Wilcoxon: p = 0.04, Fig 2C). All participants were included in AMT analysis (N = 14 per group). AMT did not statistically differ between groups and was unchanged following exercise (Table 1). Thirteen and fourteen individuals were included in the MEP_ACTIVE_ analysis for the HIGH and LOW groups, respectively. Similarly, the MEP_ACTIVE_ recruitment curves (shown in Fig 2D) and MEP_ACTIVE_ AURC were unchanged following exercise (Table 1). IPAQ scores did not correlate with the percent change in slopes for MEP_REST_ or MEP_ACTIVE_ recruitment curves (p > 0.05).

### Intracortical circuits

**Fig 3:**
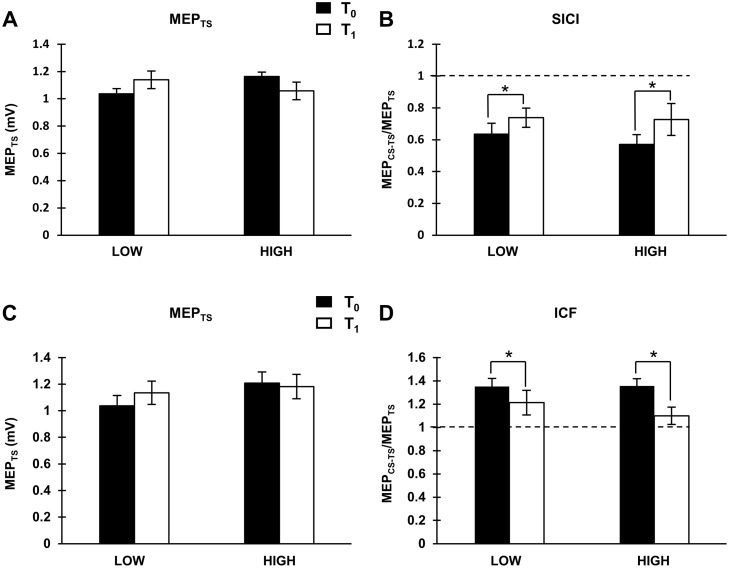
SICI: (A) Group-averaged unconditioned MEPs (i.e. MEP_TS_) (with standard errors) for both groups (LOW: N = 13; HIGH: N = 11) at both time points. (B) Group-averaged SICI (with standard errors) for each group (LOW: N = 13; HIGH: N = 11) displaying the main effect of TIME. The asterisk indicates a significant decrease in SICI. ICF: (C) Group-averaged unconditioned MEPs (i.e. MEP_TS_) (with standard errors) for both groups (LOW: N = 12; HIGH: N = 12) at both time points. (B) Group-averaged ICF (with standard errors) for each group (LOW: N = 12; HIGH: N = 12) displaying main effect of TIME. The asterisk indicates a significant decrease in ICF.

Eleven and thirteen individuals demonstrated SICI at T_0_ in the HIGH and LOW groups, respectively, and were included in subsequent analyses. Unconditioned MEPs were maintained at ~1 mV (Fig 3A). SICI was reduced following exercise regardless of physical activity level (Fig 3B, showing the main effect of TIME, Table 1, paired t-test: p = 0.007). Fifteen participants (7 HIGH, 8 LOW) demonstrated a reduction in SICI following exercise while three showed little to no change and six revealed increases in SICI. Twelve and twelve individuals demonstrated ICF at T_0_ in the HIGH and LOW groups, respectively, and were included in subsequent analyses. Unconditioned MEPs were maintained at ~1 mV (Table 1, Fig 3C). ICF was reduced following exercise regardless of physical activity level (Table 1, Wilcoxon: p = 0.04, Fig 3D). Fifteen participants (7 HIGH, 8 LOW) demonstrated a reduction in ICF following exercise while three showed little to no change and six revealed increases. SICF_1.2ms_ and SICF_2.5ms_ were not different between groups and were unchanged by exercise (Table 1).

## Discussion

This study revealed that exercise-induced short-term plasticity depends on the physical activity level of the individual. Exercise increased the amplitude of corticospinal output in the HIGH group, and in contrast, did not alter corticospinal output in the LOW group. This finding indicates that physical activity levels influence the propensity and direction of exercise-induced short-term plasticity. Our data also indicated that exercise reduces SICI, in support of previous literature [15, 16]. We extend this finding to indicate that SICI reduction occurs in both fit and relatively sedentary individuals. We discuss these findings and the implications for rehabilitation.

### Exercise induces short-term plasticity in corticospinal output

A key finding in this study is that exercise alters corticospinal excitability depending on the level of physical activity. Corticospinal excitability in the HIGH group increased by ~28% following exercise while no changes were observed for the LOW group. Our findings are similar to the effects of PAS that increases MEPs in physically active but not sedentary groups [35]. While spike-timing dependent mechanisms mediate PAS effects [47], less is known about the neural mechanisms that mediate exercise-induced plasticity. Modulation of neurotransmitter concentration may participate in increasing corticospinal excitability. A single session of aerobic exercise upregulates the release of serotonin [48, 49], norepinephrine [50, 51], and upregulates [48, 50, 52–55] or does not change [56] dopamine. These neurotransmitters have been shown to modulate the excitability of motor neurons [57–60]. Exercise-induced increases in corticospinal output may also be due to an increase in glutamate. A single session of exercise increases glutamate [57, 58], as measured via ^1^H-MRS and a positive correlation exists such that greater cortical glutamate is associated with steeper MEP recruitment curves [59].

It has been shown that long-term physical activity is linked to improved cognitive function and memory [1, 2, 5, 60]. Although the mechanisms that underpin such improvements remain unclear, there is strong evidence to implicate BDNF as a mediator of neural plasticity. Higher fitness levels are associated with a lower concentration of peripheral BDNF [61, 62], suggesting that gains in fitness may yield more efficient uptake and utilization of BDNF in the central nervous system [63]. The exercise-induced facilitation of MEPs we observed in the HIGH group may relate to an increased uptake of BDNF within the central nervous system. Future studies may consider measuring BDNF and corticospinal excitability, simultaneously.

Increases in MEPs may also be associated with the physiological differences between the two groups. The HIGH group may have increased stroke volume [12], increased brain perfusion [14], and muscle adaptions that may reduce fatigue [12]. Comparatively, the LOW group lacks these chronic adaptions to exercise, which may reduce neuroplasticity induction. This is supported by the trend towards decreases in excitability seen in the LOW group. Since we opted to control heart rate only, differences in the resting MEPs between the groups may be contributed by variances in the workload performed, stress or anxiety associated with the exercise regime and/or the rate of perceived exertion associated with the exercise intervention. Future studies will need to address whether MEPs differ between HIGH and LOW groups when the exercise intervention controls for one or more of these variables. From the present results, we can conclude that exercise controlled by heart rate revealed differences in resting MEPs only and did not create changes in intracortical circuits. However, we note again, that we cannot exclude the contribution of workload, cortisol levels, anxiety and perceived exertion, which were not controlled and may also contribute to these differences.

### Exercise induces short-term plasticity in intracortical inhibition and facilitation

We observed a reduction in SICI following exercise in support of previous literature [15, 16], but extend these findings to show that SICI is reduced irrespective of the physical activity level. Previous studies report reductions in SICI following moderate intensity exercise (~20% in [16] and ~35% in [15]). In our study, we observed an overall decrease of ~18%. The difference may reflect our less intense exercise (~60% age-predicted maximal heart rate vs. 65–70% in [15]) or the muscle studied (FDI vs. flexor carpi radialis). The mechanisms by which exercise reduces SICI may involve changes in GABA_A_ receptor activity that are considered to mediate the circuit [19, 20]. Aerobic exercise increases serum BDNF [25, 61, 63–74] that in turn reduces GABA_A_ receptor activity [75]. Additionally, in rat models, BDNF reduces GABA_A_ receptor function [76]. Thus, as suggested elsewhere [16] and above, aerobic exercise may increase serum BDNF that acts to decrease SICI.

We also observed ~15% reduction in ICF following exercise irrespective of the physical activity level. Previous study reports increase in ICF following moderate intensity exercise [15]. The disparity between previous work and our study may be attributed to differences in exercise or TMS protocols (% AMT vs. % RMT). However, our findings are similar to the effects of continuous theta burst stimulation (cTBS) over M1 which reduced both ICF and SICI [77]. ICF is thought to be mediated via glutamatergic facilitation coupled with persisting GABAergic inhibition [29]. Previous research on drugs has demonstrated that administration of GABA_A_ agonists and NMDA antagonists reduces ICF [78–80]. Further, some dopamine agonists, such as cabergoline, also reduce ICF [81] and hence, changes in ICF may be dependent on more than one neuronal circuit [82], as suggested previously [83].

### Circuitry unaltered by exercise

Several measures of cortical activity were unchanged following exercise. RMT and AMT were unaltered by physical activity levels or exercise, in support of previous literature [16, 35, 84]. Therefore, a single session of aerobic exercise does not alter the membrane thresholds. Additionally, MEP recruitment curves obtained in the actively contracted FDI were unchanged by exercise, indicating that exercise only alters corticospinal output without the voluntary activation of motor neurons. Lastly, we did not observe significant changes in the SICF circuits following exercise, suggesting that moderate intensity aerobic exercise does not modulate early and late indirect waves (i.e. I-waves), similar to other plasticity inducing paradigms [32, 33].

### Implications

One of the main implications of this work is that pre-existing physical activity levels determine the propensity for plasticity. In our sedentary group, exercise did not alter excitability, while in the active group the neuronal output to a hand muscle was enhanced, which is a major goal of rehabilitative approaches. Collectively our data suggest that physically active individuals demonstrate a propensity for increasing neuronal output to the hand muscle following a single session of exercise. We speculate that this may have ramifications for the success of rehabilitation protocols aiming to promote neural plasticity, such that individuals with greater physical activity levels may demonstrate increased propensity for plasticity. Indeed, evidence in the animal literature suggests that physically active animals show better recovery of behavioural performance than their sedentary counterparts [8]. It is important to note that exercise also protects from further neurodegeneration after injury and promotes better recovery. In humans, rehabilitation protocols involving exercise regimes have shown improvements in physical function [85], movement initiation [86], and activities of daily living [87]. Our results support the PAS results [35], indicating that short-term plasticity is observed only in the corticospinal output of physically active individuals. Therefore, regular physical activity may be a determinant for the success of rehabilitation approaches that aim to increase corticospinal output to impaired muscles. Further, our data suggests that exercise can be used to prime the motor cortex for plasticity via a reduction in inhibition, regardless of physical activity level. Recent studies have shown improvements in motor learning [88] and increased response to brain stimulation in healthy adults [24, 25, 40, 89] when exercise is used as a prime.

### Limitations and future directions

Direct assessment of aerobic capacity may be achieved using maximal volume oxygen uptake (VO_2_ max). We use the IPAQ that provides a reliable self-report of physical activity in the past 7 days [39] as used elsewhere [16, 35, 40]. Future studies may confirm our findings using VO_2_ max. Additionally, the recumbent cycle ergometer used in our study did not provide wattage as a function of resistance level. Therefore, we cannot provide the workload achieved by the exercising lower limb muscles. However, we chose to control the aerobic intensity via heart rate as performed elsewhere [15, 40], as changes in heart rate yield modification in serum BDNF [90]. An alternative approach is to control for both workload and heart rate by altering the duration of the exercise. Future studies should examine how controlling for workload or altering exercise duration affects exercise-induced neuroplasticity. We collected MEP recruitment curves using 3 pulses per intensity to reduce the intrusion of TMS-induced plasticity. This is a small number of pulses to obtain an estimate of MEP amplitudes, that while used elsewhere [91] may benefit from a greater number of stimuli delivered [92–94]. Thus, future studies should consider using more pulses per intensity to reduce variability in this dependent measure. Further, we emphasize that these findings are achieved following a single session of exercise. Multiple sessions of exercise may increase the opportunity for plasticity to be observed in sedentary individuals [11]. Finally, we have tested young adults, and it remains unknown whether these findings will also be revealed across the lifespan.

### Conclusions

The present study demonstrated that physical activity levels influence motor cortex excitability and the propensity for exercise-induced plasticity. First, corticospinal excitability is increased following exercise in highly active individuals only. Second, exercise reduces cortical inhibition regardless of physical activity level. A reduction of inhibitory input in the motor cortex creates a more favorable environment for plasticity induction. Therefore, we conclude that physical activity levels should be taken into consideration when investigating corticospinal excitability and plasticity induction within the motor cortex in healthy and clinical populations.
